# Activating HER3 mutations in breast cancer

**DOI:** 10.18632/oncotarget.25576

**Published:** 2018-06-12

**Authors:** Rosalin Mishra, Samar Alanazi, Long Yuan, Thomas Solomon, Tarjani M. Thaker, Natalia Jura, Joan T. Garrett

**Affiliations:** ^1^ James L. Winkle College of Pharmacy, University of Ohio, Cincinnati, Ohio, USA; ^2^ Department of Cellular and Molecular Pharmacology, Cardiovascular Research Institute, University of California, San Francisco, California, USA

**Keywords:** mutation, HER2, HER3, ER, Targeted Therapy

## Abstract

Recent studies have highlighted a role of HER3 in ER and HER2-driven breast cancers. We sought to investigate the role of patient-derived HER3 mutations in ER+ and HER2+ breast cancer cells using ectopic expression of HER3 mutants. We found that HER3^T355I^ mutant is activating with increased cell proliferation in ER+ T47D and MCF-7 breast cancer cells lacking HER2 over-expression. Immunoblotting and receptor tyrosine kinase array results indicated that T47D and MCF-7 cells expressing HER3^T355I^ had increased p-HER4 and p-HER1 expression. Our data showed that HER3^T355I^ induced cell proliferation is via HER4/HER1-dependent ERK1/2 and cyclinD1 mediated pathways in ER+ cells. ERα expression is upregulated in ER+ cells expressing HER3^T355I^ mutant. We noted crosstalk between ERα and HER3 in T47D cells. Several HER3 mutants (F94L, G284R, D297Y, T355I, and E1261A) acquired a gain-of-function phenotype in MCF10AHER2 cells and were resistant to lapatinib. These mutants increased HER2-HER3 heterodimerization. Knocking down HER3 from ovarian and colorectal cancers with endogenous HER3 mutations abrogated cancer cell proliferation. Overall, this study provides the first systematic assessment of how mutations in HER3 affect response of ER+ and HER2+ breast cancers to clinically relevant inhibitors and finds that HER3 mutations can be activating independent of HER2 over-expression.

## INTRODUCTION

HER3 (ERBB3) is a member of human epidermal growth factor receptor (EGFR) family. It contains an extracellular domain (ECD), a transmembrane and an intracellular domain. HER3 has significantly impaired intrinsic kinase activity and transduces signals in the presence of a ligand via forming heterodimers with other members of the EGFR family, EGFR (HER1), HER2, HER4 [1]. In addition, HER3 is described to signal in complexes with several other receptor tyrosine kinases (RTKs) such as fibroblast growth factor receptor 2 (FGFR2) and hepatocyte growth factor receptor (HGFR) [2]. Neuregulin-1 and Neuregulin-2 are high affinity ligands for HER3. In the absence of ligand, HER3 sub-domain arm II is locked in a tethered, auto-inhibitory configuration refraining from forming homo- or heterodimers [3]. Ligand binding breaks the tether and allows HER3 to adopt an extended conformation, enabling heterodimerization with other receptors.

HER3 is emerging as an important molecule in estrogen receptor (ER)+ breast cancers, which accounts for about 80% of all breast cancers [4]. HER3 mRNA is highest in ER+ or luminal tumors [5, 6], consistent with the observation that HER3 is required for cell survival in the luminal but not the basal normal mammary epithelium [7]. Treatment with the ER downregulator fulvestrant induces protein expression and activity of HER3 in ER+ breast cancer cells *in vitro*. This upregulation of HER3 drives neuregulin-mediated resistance to fulvestrant [8]. The use of HER3 neutralizing antibodies in combination with anti-estrogen treatment results in decreased tumor cell growth and delays resistance [6, 9, 10].

About 20% of all breast cancers diagnosed are HER2 positive (+) [11]. HER3 is as essential as HER2 for maintaining cell viability in HER2-overexpressing breast cancer cells [12]. HER2 is unable to directly bind and activate p85, the regulatory subunit of PI3K. HER3 contains six p85-binding motifs and when dimerized with and activated by HER2, it can potently activate PI3K signaling. Inhibition of the HER2 tyrosine kinase (TK) results in upregulation of HER3 transcription and HER3 phosphorylation. This compensatory phosphorylation of HER3 partially maintains PI3K/AKT signaling [13]. These results suggest that HER2-dependent breast cancers rely on HER3 to drive their growth and survival.

HER3 is mutated in 1-3% of primary [14, 15] and up to 14% of metastatic ER+ breast cancers [16]. HER3 somatic mutations occur in several cancers including colon, lung, gastric, ovarian, glioblastomas [17, 18]. Jaiswal *et al.* reported that several HER3 mutants transformed colonic and breast cancer cells in a ligand-independent manner. Mutant HER3-mediated oncogenic activity is dependent on HER2 and is curtailed both *in vitro* and *in vivo* using agents that either target HER3 directly or indirectly [18].

In this study, using various *in vitro* models, we have delineated molecular mechanisms by which patient-derived HER3 T355I mutant activates ER+ T47D and MCF-7 cells. We also show that several HER3 mutants acquire a gain-of-function phenotype in HER2 overexpressing MCF10A cells. Our findings suggest certain HER3 mutants are oncogenic in the absence or presence of HER2 over-expression.

## RESULTS

### HER3 mutations are proliferative and activate MAPK and HER signaling in ER+ breast cancer cells

**Figure 1:**
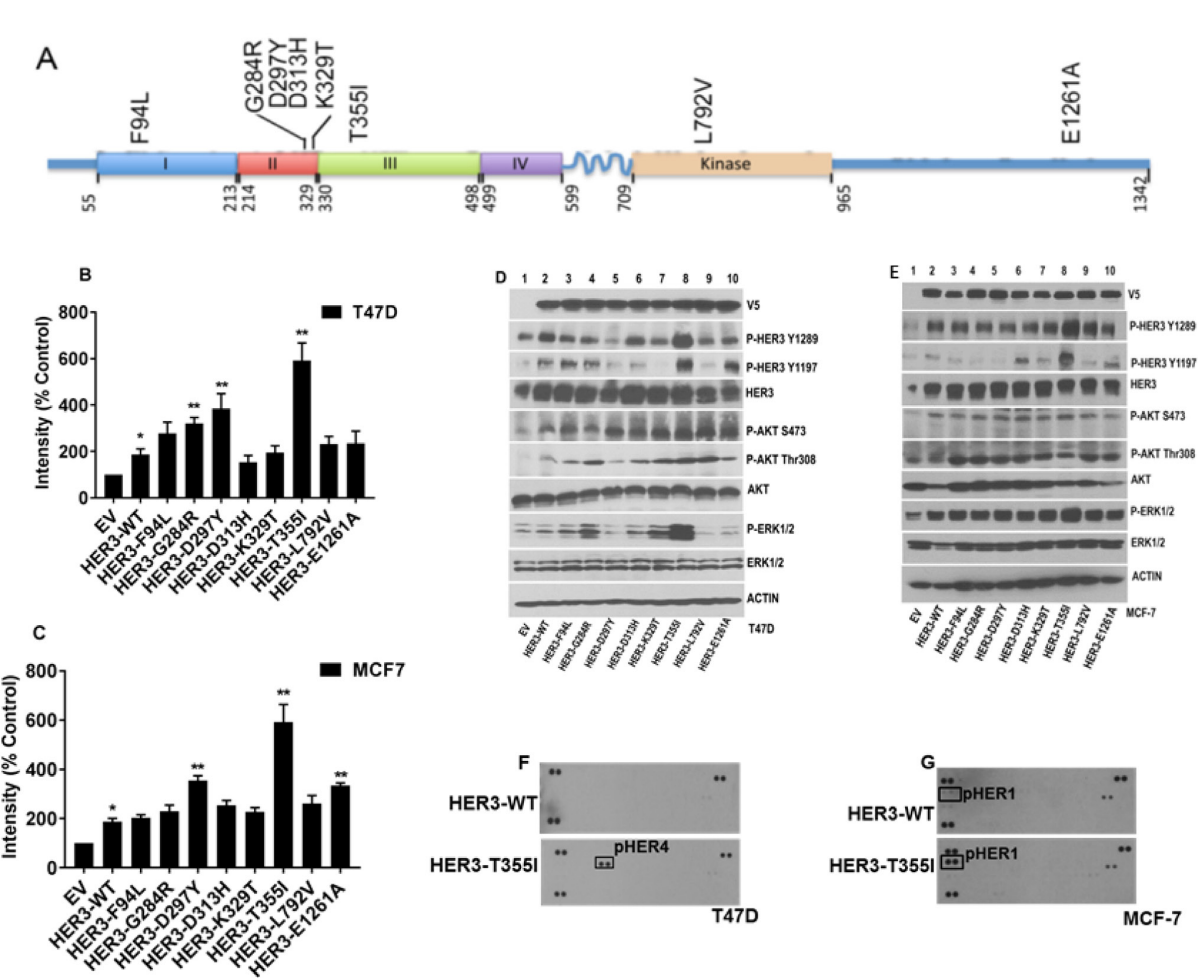
Oncogenic potential of ER+ cells expressing HER3 mutations (**A**) HER3 nonsynonymous somatic mutations studied in this work depicted over HER3 protein domain. (**B**) T47D cells expressing HER3^EV^, HER3^WT^ and HER3 mutants (F94L, G284R, D297Y, D313H, K329T, T355I, L792V, and E1261A) were plated, treated and stained with crystal violet as described in Materials and Methods. Intensities were represented as mean; Error bars: SEM (*n =* 3 independent experiments performed in triplicate),^*^P < 0.05 versus EV and ^**^P < 0.05 versus WT. (**C**) MCF-7 cells with HER3 constructs were plated and stained under above similar conditions. The value is represented as mean of intensities; Error bars: SEM (*n =* 3 independent experiments performed in triplicate). ^*^P < 0.05 versus EV and ^**^P < 0.05 versus WT. (**D–E**) Signaling pathways in serum starved T47D and MCF-7 cells expressing HER3 EV, WT and mutants determined by western blot. Actin served as loading control. (**F–G**) RTK arrays used to determine tyrosine phosphorylation of various RTKs from lysates of T47D and MCF-7 cells expressing HER3^WT^ and HER3^T355I^ serum starved overnight.

We aimed to distinguish between HER3 mutations that drive cancer progression versus passenger mutations in breast cancer. Passenger mutations are not thought to contribute to cancer growth; rather, they simply accrue during the course of tumor development as a result of genomic instability. We analyzed the oncogenic potential of 8 patient-derived HER3 missense mutations (F94L, G284R, D297Y, D313H, K329T, T355I, L792V, and E1261A). 6 HER3 mutations (F94L, G284R, D297Y, D313H, K329T, and T355I) were identified in the ECD, HER3^L792V^ in the kinase domain, and HER3^E1261A^ in the intracellular tail of HER3 (Figure 1A). Details of patient HER2/ER status whose tumor harbour a HER3 mutation are listed in Supplementary Table 1. ER/HER2 expression status was evaluated using the indicated techniques (Supplementary Table 1). HER2 expression is analyzed in MCF7 and T47D cells using western blot (Supplementary 1D) and do not over-express HER2 [19]. Since HER3 is mutated in up to 14% of metastatic ER+ breast cancers [16], we introduced the above HER3 mutations along with HER3^EV^ (EV- empty vector) and HER3^WT^ (WT- wild-type) into ER+ T47D and MCF-7 cells using lentiviral transduction as described in Materials and Methods. We confirmed the stable transduction of HER3 by V5-tagged protein expression (Figure 1D and 1E). Proliferation assays revealed that HER3^D297Y^ and HER3^T355I^ have increased proliferation in both ER+ cells compared to other mutants (Figure 1B and 1C, Supplementary Figure 1A and 1B). HER3^T355I^ had elevated levels of p-HER3, p-ERK1/2 in both T47D and MCF-7 cells. In addition, we observed increased AKT phosphorylation at Ser473 and Thr308 in T47D cells with HER3^T355I^ versus HER3^WT^. We did not observe increased AKT activation in MCF7 cells expressing HER3^T355I^ versus HER3^WT^. We did not observe significant increased activation of HER3, AKT or ERK1/2 in ER+ cells expressing HER3^D297Y^ compared to cells with HER3^WT^ (Figure 1D and 1E). Since we observed increased proliferation and activation of downstream signaling in cells expressing HER3^T355I^ versus HER3^WT^, we wished to examine the phosphorylation status of 49 receptor tyrosine kinases (RTK) and 43 kinases using commercially available antibody arrays. The phosphokinase array confirmed activation of the MAPK pathway in T47D cells with HER3^T355I^ versus HER3^WT^ (Supplementary Figure 1C). HER3 heterodimerizes and activates the EGFR family members EGFR and HER4 [20, 21]. Interestingly, our RTK array data revealed that serum starved cells with HER3^T355I^ expression have activated HER4 in T47D and HER1 in MCF-7 cells versus HER3^WT^ (Figure 1F and 1G). Western blotting data also indicated that T47D and MCF-7 cells expressing HER3^T355I^ has elevated p-HER4 and p-HER1 versus HER3^WT^ (Figure 3A and Figure 3C). This suggested that HER3^T355I^ might signal via a HER4/HER1-dependent mechanism in ER+ cells.

**Figure 2:**
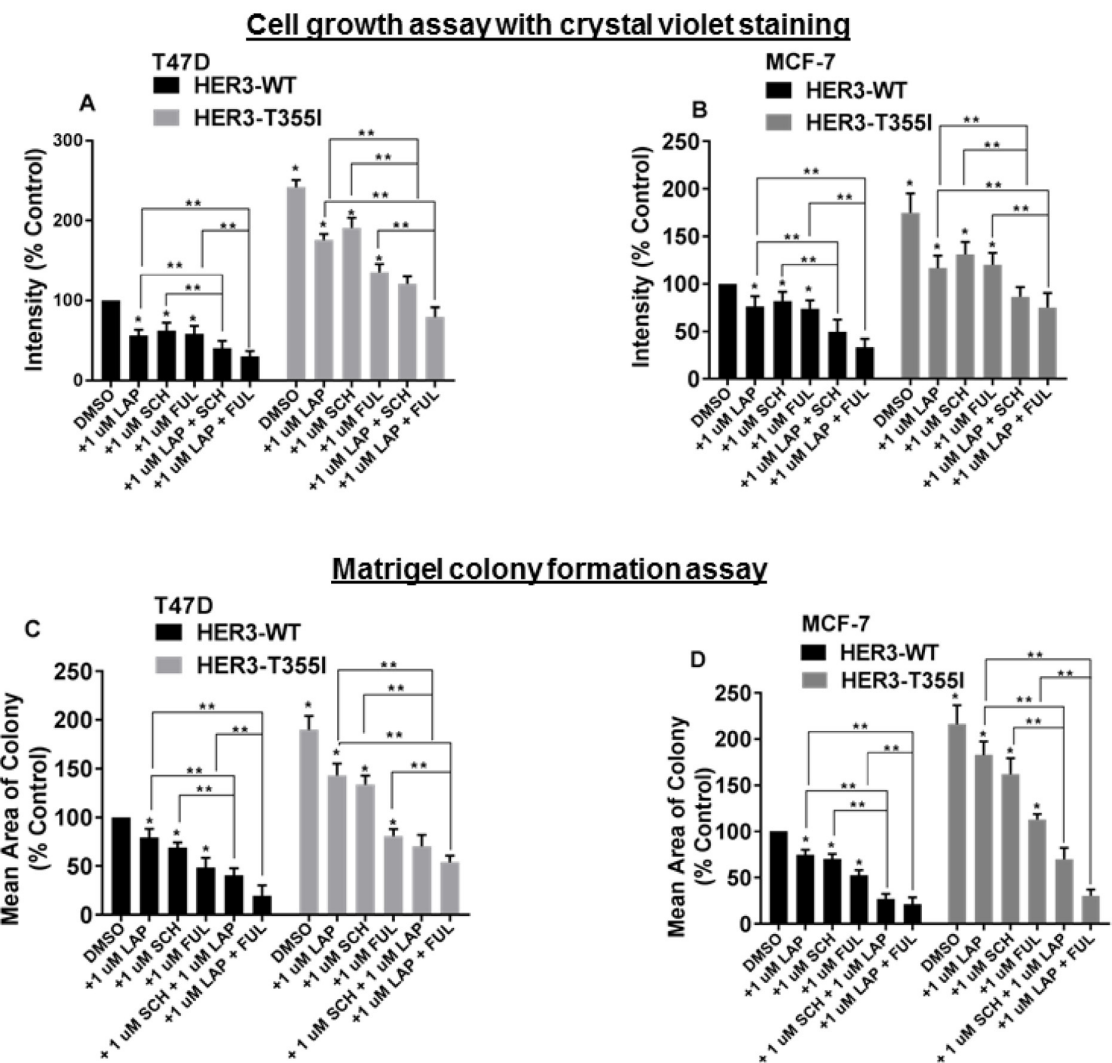
Blocking HER1/HER4, ER and ERK1/2 signaling inhibits the proliferation of ER+ cells with HER3^T355I^ (**A**) T47D cells (HER3^WT^ and HER3^T355I^) were plated and treated with vehicle (DMSO), lapatinib (1 µM), fulvestrant (1 µM), SCH772984 (1 µM), or indicated combinations. Media and inhibitors were replenished every second day and stained with crystal violet. The value is represented as mean ± SEM (*n =* 3 independent experiments performed in triplicate). ^*^P < 0.05 versus WT, ^**^P < 0.05 versus treated groups as indicated. (**B**) MCF-7 cells (HER3^WT^ and HER3^T355I^) were plated and treated with vehicle (DMSO), lapatinib (1 µM), fulvestrant (1 µM), SCH772984 (1 µM) or indicated combinations. Intensities were analyzed and represented as mean ± SEM (*n =* 3 independent experiments performed in triplicate). ^*^P < 0.05 versus WT, ^**^P < 0.05 versus respective treatment groups as shown. (**C–D**) ER+ T47D and MCF-7 cells with HER3^WT^ and HER3^T355I^ expression were seeded on a basement membrane of matrigel ± vehicle (DMSO), lapatinib (1 µM), fulvestrant (1 µM), SCH772984 (1 µM) or indicated combinations. The average size of each cellular structure was quantified using ImageJ and expressed relative to respective control. The value is represented as mean of areas ± SEM (*n =* 3 independent experiments performed in triplicate). ^*^P < 0.05 versus WT, ^**^P < 0.05 versus respective treatment groups as indicated.

**Figure 3:**
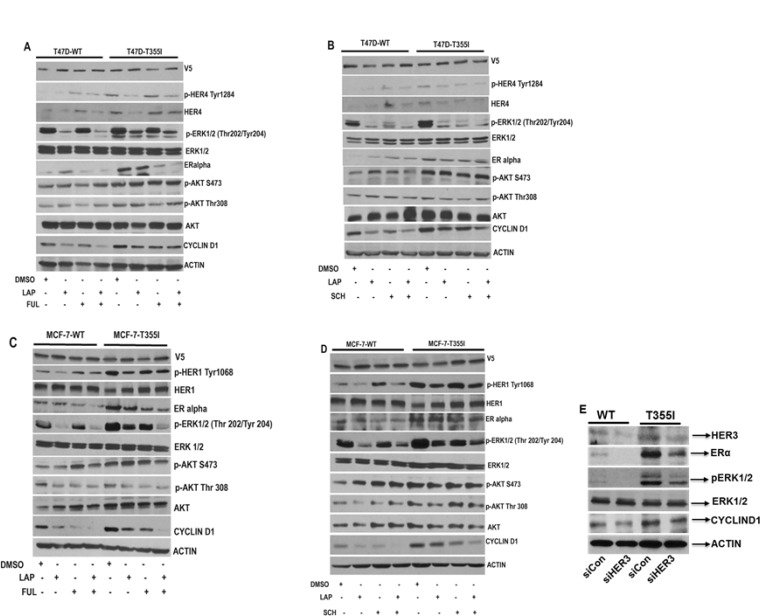
Abrogation of HER1/HER4 and ERK1/2 signaling inhibits cyclinD1 expression in ER+ cells with HER3^T355I^ (**A–B**) Serum starved T47D cells expressing HER3^WT^ and HER3^T355I^ were treated with vehicle (DMSO), lapatinib (1 µM), fulvestrant (1 µM), SCH772984 (1 µM) or indicated combinations for 4 hr and cell lysates were collected and immunoblotted using p-HER4, p-ERK1/2, ERα, p-AKT and cyclinD1 antibodies. Actin served as loading control. (**C–D**) Serum starved MCF-7 cells (HER3^WT^ and HER3^T355I^) were treated with vehicle (DMSO), lapatinib (1 µM), fulvestrant (1 µM), SCH772984 (1 µM) or indicated combinations for 4 hr and cell lysates were collected and immunoblotted using indicated antibodies. (**E**) T47D cells with HER3^WT^ and HER3^T355I^ expression were transfected with an HER3-specific siRNA or control (siCon) for 48 hr and analyzed using specific antibodies. Actin was used as loading control.

### Abrogating HER1/HER4, ERK1/2 and ER signaling suppresses HER3^T355I^ induced proliferation in ER+ cells

Data indicated that MAPK (p-ERK1/2) and HER4/HER1 pathways are activated in ER+ cells expressing HER3^T355I^ (Figure 1D–1G). Lapatinib inhibits all active HER kinases [22, 23]. Hence, T47D and MCF-7 cells with HER3^WT^ and HER3^T355I^ expression were treated with lapatinib in the presence or absence of the ER inhibitor fulvestrant and the ERK1/2 inhibitor SCH772984 to analyze the effect on cell growth. The data indicated that combined treatment of lapatinib and fulvestrant reduced cell proliferation of HER3^WT^ and HER3^T355I^ significantly versus individual treatments in both ER+ cells. We also observed a prominent reduction in growth kinetics of these cells when subjected to co-treatment of lapatinib and SCH772984 as compared to individual agents (Figure 2A, 2B and Supplementary Figure 2A and 2B). We performed further studies in a reconstituted basement membrane of matrigel as shown in Figure 2C and 2D. We noted significant reduction in acini formation in cells expressing HER3^WT^ and HER3^T355I^ when subjected to co-treatment of lapatinib and fulvestrant or lapatinib and SCH772984 as compared to individual drugs (Figure 2C, 2D and Supplementary Figure 3A and 3B). The combined effect of 1 µM lapatinib + 1 µM SCH772984 or 1 µM lapatinib + 1 µM fulvestrant was synergistic in both ER+ cells as indicated by combination index (CI) values (Supplementary Figure 2C and 2D). Overall, these data indicate that inhibition of HER and ER signaling is sufficient to reduce the proliferation of ER+ cells expressing HER3^T355I^.

### CylinD1 mediates signaling downstream of HER4/ERK1/2 in T47D and HER1/ERK1/2 in MCF-7 cells expressing HER3^T355I^

We attempted to further delineate whether a HER/MAPK-dependent mechanism triggered the increased proliferation of HER3^T355I^. Therefore, T47D and MCF-7 cells with HER3^WT^ and HER3^T355I^ expression were subjected to lapatinib treatment in the presence or absence of fulvestrant and SCH772984. Our western blot data demonstrated that lapatinib inhibited the activation of HER4 and HER1 respectively in T47D and MCF-7 cells with HER3^WT^ and HER3^T355I^ expression (Figure 3A–3D). It is reported that fulvestrant induces HER3/HER4 expression in ER+ cells [8]. Consistent with this, a partial upregulation of HER4 expression was observed in response to fulvestrant treatment in T47D^WT^ cells (Figure 3A). Lapatinib alone inhibited p-ERK1/2 in both T47D and MCF-7 cells with HER3^WT^ and HER3^T355I^ expression (Figure 3A–3D). SCH772984 as single agent reduced ERK1/2 activation in both ER+ cells with HER3^WT^ and HER3^T355I^ expression. Co-treatment with lapatinib and SCH772984 resulted in similar p-ERK1/2 levels compared to individual lapatinib or SCH772984 treatment (Figure 3B and Figure 3D). There was no significant alteration in AKT signaling in response to individual inhibitors or combined treatment (Figure 3A and 3C). This is consistent with findings by Emde *et al.* who reported that co-treatment of fulvestrant and lapatinib have no effect on AKT activation in ER+ breast cancer cells [24]. Interestingly, we observed that lapatinib and SCH772984 alone or in combination downregulated cyclin D1 expression in T47D and MCF-7 cells expressing HER3^WT^ and HER3^T355I^ (Figure 3B and Figure 3D). Fulvestrant also suppressed cyclin D1 expression in both cells with HER3^WT^ and HER3^T355I^. Fulvestrant in combination with lapatinib had a more prominent effect in reducing cyclin D1 expression in MCF-7 compared to T47D cells with HER3^WT^ and HER3^T355I^ expression (Figure 3A and 3C). We observed an upregulation of ERα expression in ER+ cells with HER3^T355I^ versus HER3^WT^ (Figure 3A–3D). Our data indicated that lapatinib partially restores ERα expression in T47D cells with HER3^WT^ (Figure 3A–3B). Kronblad *et al.* demonstrated that inhibition of ERK1/2 restored ERα expression in ER+ breast cancer cells [25]. Our results indicated that SCH772984 partially restored ERα expression in T47D cells expressing HER^WT^ (Figure 3B). Furthermore, we knocked down HER3 in T47D cells using HER3 specific siRNA and observed a significant downregulation of ERα, p-ERK1/2 and cyclin D1 expression (Figure 3E), indicating that silencing HER3 expression inhibits downstream signaling.

### Transition of HER3^T355I^ from inactive to active confirmation

**Figure 4:**
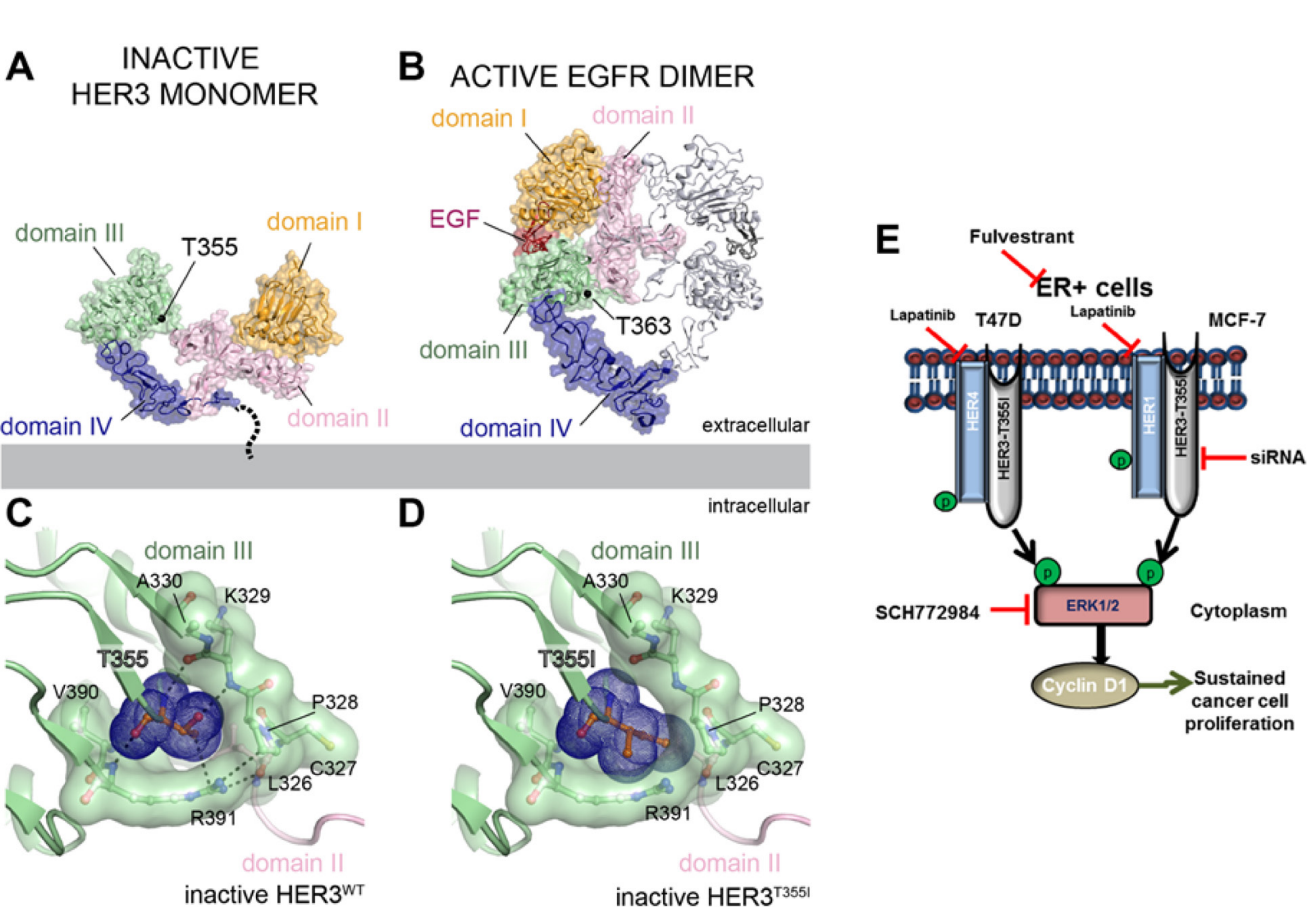
Structural analysis of HER3^T355I^ mutation (**A-B**) The locations of the conserved T355 residue mapped onto the structure of the inactive HER3 extracellular domain (ECD) monomer (PDB ID: 1M6B) (**A**) and the active EGFR ECD homodimer (PDB ID: 3NJP) (**B**) illustrating the conformational changes associated with ligand dependent receptor activation. In EGFR, domains I and II rotate around the domain II/III hinge as a single rigid body to form an extended, activated conformation poised to interact with dimerization partners. For clarity, one monomer of the EGFR ECD homodimer is shown using the same surface representation as for the inactive HER3 ECD monomer. The second monomer is shown as grey ribbons. (**C**) Cartoon representation of interactions between T355 and residues of the hinge region pocket in the inactive HER3 ECD. (**D**) Cartoon representation of the modelled isoleucine residue at position 355 (I355) in the HER3 ECD reveals that the bulkier hydrophobic residue produces a steric clash with the polar pocket. (**E**) Schematic representation of HER4/HER1-activated ERK1/2-dependent and cyclinD1-mediated signaling in ER+ T47D and MCF-7 cells expressing HER3^T355I^ mutant.

We next performed structural modeling to gain insight into the mechanism behind the activating effect of the T355I mutation in HER3. T355I is located at the base of domain III of the ECD near a hinge between domains II and III (Figure 4A). Based on studies on EGFR, all ligand-binding HER receptors, including HER3, have been proposed to undergo a conformational change upon ligand binding where domains I and II rotate around the hinge as a single rigid body to form an extended, activated conformation poised to interact with dimerization partners (Figure 4B) [26]. Analysis of the inactive structure of the HER3 ECD (PDB ID: 1M6B) reveals that a number of charged and polar residues within the domain II/III hinge form a pocket that accommodates T355. Interactions between T355 and residues within the pocket likely stabilize HER3 in the inactive, tethered conformation by restraining the receptor from rotating around the domain II/III hinge in the absence of ligand-dependent activation (Figure 4C). Mutation of T355 to a bulkier hydrophobic isoleucine (355I) is expected to produce a steric clash with the polar pocket in the tethered, unliganded state (Figure 4D), favouring the extended, active conformation observed for the activated EGFR homodimeric ECD complex (Figure 4B). Thus, T355I mutation might promote HER3 signaling by shifting the conformational equilibrium of the receptor to an extended conformation, even in the absence of a ligand. Although at present, there is no structure of HER3 ECD in the extended conformation, analysis of the ligand-bound EGFR ECD (PDB ID: 3NJP) in which the analogous threonine is conserved (T363), shows that T363 does not directly engage residues of the dimerization interface (Figure 4B). This further supports the role of the T355I mutation in disruption of HER3 autoinhibition rather than stabilizing an active dimeric complex. Figure 4E represents HER4/HER1-dependent ERK1/2- and cyclinD1-mediated cell proliferation of HER3^T355I^ in ER+ T47D and MCF-7 cells.

### HER3 mutations are oncogenic in MCF10AHER2 cells and induce stable heterodimer with HER2

**Figure 5:**
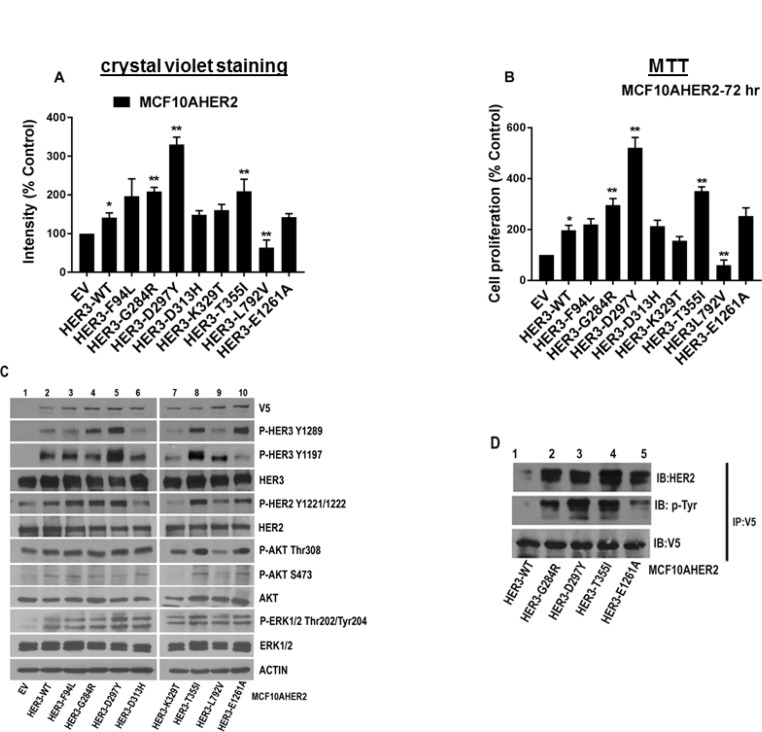
Oncogenic potential of MCF10AHER2 cells expressing HER3 mutations (**A**) MCF10AHER2 cells expressing HER3^EV^, HER3^WT^ and mutants (F94L, G284R, D297Y, D313H, K329T, T355I, L792V, and E1261A) were plated. Media with 2 µg/ml of puromycin was replenished every second day and stained with crystal violet. The intensities are represented as mean; Error bars: SEM (*n =* 3 independent experiments performed in triplicate). ^*^P < 0.05 versus EV and ^**^P < 0.05 versus WT. (**B**) Growth kinetics of MCF10AHER2 cells with HER3 mutations was determined *in vitro* after 72 hr and represented in form of bar graph. Error bars: SEM, (*n =* 3 independent experiments performed in triplicate).^*^P < 0.01 versus EV, ^**^P < 0.05 versus WT. (**C**) Signaling in MCF10AHER2 cells expressing HER3 mutations assessed by western blot. Whole cell extracts isolated from MCF10AHER2 cells were examined using antibodies described in Materials and Methods. Actin was used as the loading control. (**D**) MCF10AHER2 WT or mutants (G284R, D297Y, T355I and E1261A) cells were immunoprecipitated using a V5 antibody. An immunoprecipitation assay was performed as described in Materials and Methods and the products were analyzed by 7.5% SDS-PAGE followed by V5, p-Tyr and HER2 immunoblots.

We next wished to examine the role of HER3 mutations in a model of HER2 overexpressing mammary epithelial cells. Accordingly, MCF10AHER2 cells were transduced with HER3 EV, WT and mutant constructs and stable cell lines were generated. We identified several HER3 mutations (F94L, G284R, D297Y, T355I, and E1261A) with higher cell proliferation than HER3^WT^ (Figure 5A, Figure 5B and Supplementary Figure 4A and 4B). MCF10AHER2 cells with HER3^G284R^ had elevated levels of p-HER3 (Y1289) and p-HER2 (Y1221/1222) versus HER3^WT^. HER3^D297Y^ significantly maintained enhanced levels of p-HER3 (Y1289/Y1197), p-HER2 (Y1221/1222) and p-ERK1/2 levels versus HER3^WT^. HER3^T355I^ showed significant phosphorylation of HER3 (Y1289/Y1197) and HER2 (Y1221/1222) versus HER3^WT^. HER3^E1261A^ showed only elevated p-HER3 (Y1289) versus HER3^WT^. MAPK (ERK1/2) pathway was not significantly activated in HER3 mutants (F94L, G284R, T355I and E1261A) except HER3^D297Y^. There was no significant alteration in p-AKT levels (Figure 5C). These data are consistent with findings by Jaiswal *et al.* [18]. We investigated if there was more HER2 bound to mutant HER3 compared to HER3^WT^. Immunoprecipitation data confirmed the presence of significant HER2-HER3 dimers in MCF10AHER2 cells expressing HER3 mutants compared to WT control (Figure 5D). Therefore, we targeted HER3’s heterodimeric partner, HER2 using monoclonal antibodies, trastuzumab and pertuzumab which are directed against HER2 dimerization domain IV and II respectively. Matrigel growth assay data revealed that cells expressing HER3^WT^ were not sensitive to trastuzumab nor pertuzumab or the combination. Interestingly, HER3^D297Y^ was sensitive and had reduced colony formation in presence of pertuzumab, trastuzumab or combination of both (Supplementary Figure 4C and 4D).

### MCF10AHER2 cells expressing HER3 mutants exhibit resistance to lapatinib

**Figure 6:**
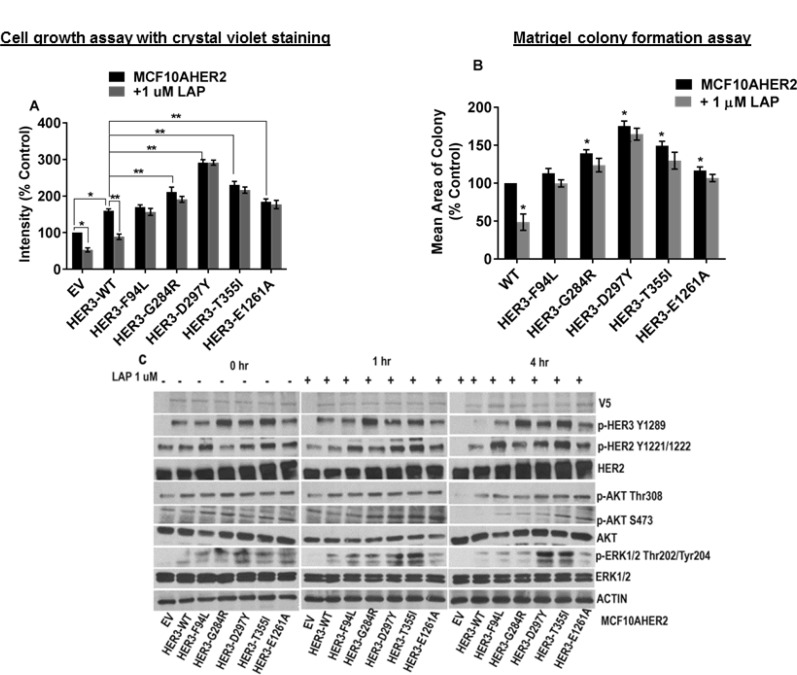
MCF10AHER2 cells expressing HER3 mutations exhibit resistance to lapatinib (**A**) MCF10AHER2 cells expressing HER3^EV^, HER3^WT^ and HER3 mutants (F94L, G284R, D297Y, T355I and E1261A) were treated ± 1 µM lapatinib. Media with vehicle and drugs were replenished every second day and stained. The value is represented as mean of intensities ± SEM, (*n =* 3 independent experiments performed in triplicate). ^*^P < 0.001 versus EV without lapatinib treatment, ^**^P < 0.05 versus WT without lapatinib treatment. (**B**) MCF10AHER2 cells with HER3^WT^ or mutants (F94L, G284R, D297Y, T355I and E1261A) were seeded on a basement membrane of matrigel ±1 µM lapatinib. The average size of each cellular structure was quantified using ImageJ and expressed relative to respective control. The value is represented as mean of areas; Error bars: SEM,(*n =* 3 independent experiments performed in triplicate).^*^P < 0.05 versus WT without lapatinib treatment (**C**) Serum starved MCF10AHER2 cells expressing HER3^EV^, HER3^WT^ and HER3 mutants (F94L, G284R, D297Y, T355I and E1261A) were treated with lapatinib (1 µM) for 1 and 4 hr, total protein was extracted and the levels were examined using the indicated antibodies.

Lapatinib is a potent ATP-competitive HER2 tyrosine kinase inhibitor (TKI). Hence, we examined if HER3 mutants expressed in MCF10AHER2 cells confer resistance to lapatinib. The results indicated that lapatinib did not suppress cell proliferation induced by HER3 mutants (F94L, G284R, D297Y, T355I, and E1261A) versus respective vehicle controls. However, HER3^EV^ and HER3^WT^ showed significant reduction in cell proliferation in the presence of lapatinib (Figure 6A, Supplementary Figure 5A). Several HER3 mutants (G284R, D297Y and T355I) induced significantly large acini on matrigel (Supplementary Figure 5B). Lapatinib did not block acini formation by HER3 mutants. However, colony formation was markedly reduced in HER3^WT^ in response to lapatinib treatment (Figure 6B and Supplementary Figure 5B). Western blotting results indicated that lapatinb did not affect the activation of HER2, HER3 and ERK1/2 in HER3 mutants (F94L, G284R, D297Y, T355I and E1261A) but suppressed p-HER2, p-HER3 and p-ERK1/2 levels in HER3^WT^ control (Figure 6C).

### Sensitivity of MCF10AHER2 cells expressing HER3 mutants to neratinib

**Figure 7:**
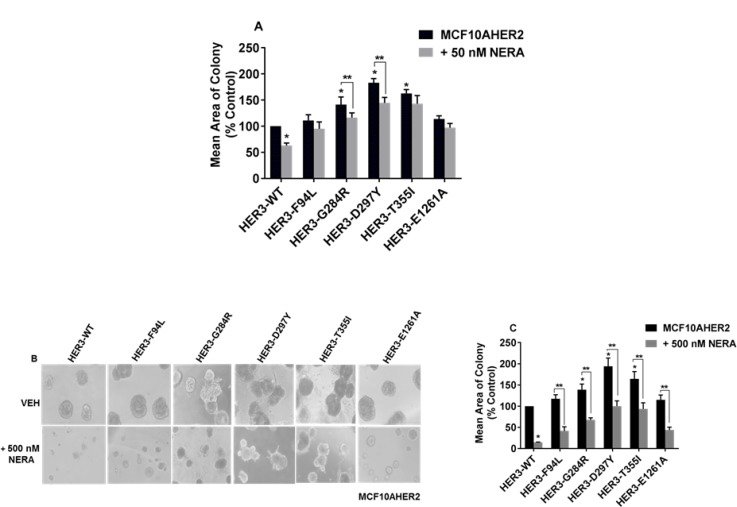
Sensitivity of MCF10AHER2 cells expressing HER3 mutations to neratinib (**A**) MCF10AHER2 cells expressing HER3^WT^ and HER3 mutants (F94L, G284R, D297Y, T355I and E1261A) were seeded on a basement membrane of 3D matrigel ± 50 nM neratinib. The average size of each cellular structure was quantified. The value is represented as mean of areas ± SEM, (*n =* 3, from three independent experiments. ^*^P < 0.01 versus WT without neratinb treatment, ^**^P < 0.05 versus respective HER3 mutants (G284R and D297Y) without neratinb treatment. (**B**–**C**) MCF10AHER2 cells expressing HER3^WT^ and HER3 mutants (F94L, G284R, D297Y, T355I and E1261A) were seeded on a basement membrane of matrigel and treated with 500 nM neratinib every second day. Phase contrast images of acini were captured at 10x magnification and the average size of each cellular structure was quantified and expressed as mean of areas ± SEM, (*n =* 3 independent experiments performed in triplicate). ^*^P < 0.05 versus WT without neratinib treatment and ^**^P < 0.01 versus respective HER3 mutants (F94L, G284R, D297Y, T355I and E1261A) without neratinib treatment.

Neratinib is an irreversible TKI used to treat HER2+ breast cancer patients [27]. Bose *et al.* reported that a minimal concentration of neratinib (<50 nM) is effective against HER2 mutants in MCF10AHER2 cells [28]. Therefore, we examined whether HER3 mutants were sensitive to neratinib. Our data demonstrated that 50 nM neratinib was not significant to suppress colony formation induced by HER3^F94L^, HER3^T355I^ and HER3^E1261A^ on matrigel (Figure 7A and Supplementary Figure 5C). A higher concentration of neratinib (500 nM) was effective in abrogating colony formation induced by most HER3 mutants (Figure 7B and Figure 7C).

### Knocking down HER3 inhibits cell proliferation in cancers harboring endogenous HER3 mutations

**Figure 8:**
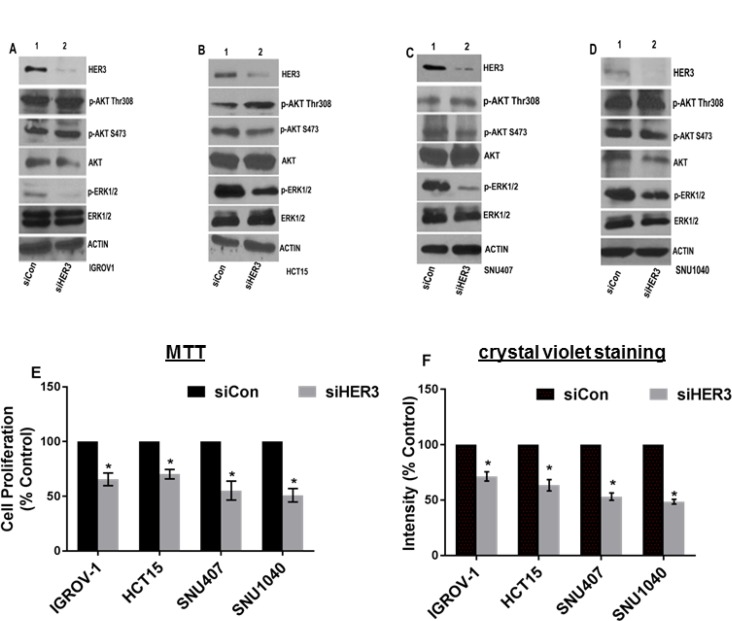
Knocking down HER3 inhibits cell proliferation in various cancers harboring endogenous HER3 mutations (**A**) Ovarian cancer cell line (IGROV-1) with HER3^K742E^ was transfected with siRNA targeting HER3 (siHER3) or a non-silencing control (siCon) and immunoblotted using HER3 antibody. Levels of HER3, p-AKT, p-ERK1/2 were analyzed. Actin was used as loading control. (**B–D**) Levels of HER3 and other indicated antibodies were analyzed in colon cancer cell lines harboring endogenous HER3 mutation (HER3^N126K^/^R667H/P1142H^ in HCT15, HER3^P262H^/^V104M^ in SNU407 & HER3^A232V/H374Y/R683L/P1162L/R1309H^ in SNU1040). Actin served as loading control. (**E**) Cells were transfected using HER3 siRNA or control siRNA and seeded to analyze for cell proliferation. Growth kinetics was analyzed using MTT assay and represented in form of bar graph. Error bars: SEM; (*n =* 3 independent experiments performed in triplicate); ^*^P < 0.05 versus siCon for various cell lines. (**F**) Ovarian and colorectal cancer cell lines were transfected with siHER3 or siCon and reseeded and stained with crystal violet. Intensities were analyzed and represented using bar graph. The value represented as mean of intensities; Error bars: SEM (*n =* 3 independent experiments performed in triplicate); ^*^P < 0.05 versus ConsiRNA.

The HER3^K742E^ mutation occurs endogenously in IGROV-1 (ovarian) and HER3^P262H^/^V104M^ in SNU-407 (colorectal), HER3^A232V/H374Y/R683L/P1162L/R1309H^ in SNU-1040 (colorectal), HER3^N126K^/^R667H/P1142H^ in HCT-15 (colorectal) cancer cell lines [29]. We proposed to study the effect of knocking down HER3 in these cancer cells. Western blot data indicated that knocking down HER3 using HER3-specific siRNA resulted in significant reduction in p-ERK1/2 expression with no alteration in AKT pathway versus control in ovarian and colorectal cancer cell lines (Figure 8A–8D). Cell growth assays using MTT and crystal violet staining indicated that each cell line had a reduction in proliferation with knockdown of endogenous HER3 (Figure 8E, Figure 8F and Supplementary Figure 6A–6D). These data further suggest the potential for HER3 mutations to be oncogenic.

## DISCUSSION

HER3 is a part of an intricate signaling network consisting of the PI3K/AKT, Ras/Raf/MAPK, JAK/STAT and PKC pathways. There is evidence that HER3 driver mutations render resistance to EGFR- and HER2-targeted therapies [18]. In this study, we expressed HER3 mutations (F94L, G284R, D297Y, D313H, K329T, T355I, L792V, and E1261A) in ER+ T47D and MCF-7 breast cancer cells. We identified HER3^T355I^ to be markedly proliferative in ER+ breast cancer cells.

Amino acid threonine 355 of HER3 has been identified as a recurrent hotspot (statistically significant) in a population-scale cohort of tumor samples of various cancer types [30]. Several large-scale cancer genomics data sets reveal tumors with the presence of a mutation at T355 of HER3 including cervical squamous cell carcinoma (*n =* 1 of 310), bladder urothelial carcinoma (*n =* 1 of 413), stomach adenocarcinoma (*n =* 1 of 559), colon adenocarcinoma (*n =* 1 of 640), ampullary carcinoma (*n =* 1 of 160). The frequency of mutation at T355 in HER3 ranges from 0.156% to 0.625% of tumors sequenced [30]. In line with other cancer types, 5 of 3614 breast cancer tumors from the METABRIC and TCGA cohorts contain a mutation at T355 with a frequency of 0.138%. All these breast cancer patients are ER+ (Supplementary Table 1). Furthermore, 2 of these tumors lack HER2 over-expression signifying the clinical relevance of studying the HER3 T355I mutation in ER+ breast cancer models lacking HER2 overexpression.

We report a novel upregulation of ERα expression in ER+ cells expressing HER3^T355I^ versus HER3^WT^. Collins *et al.* found ERα and HER3 co-immunoprecipitated when HEK293 cells were co-transfected with ERα and HER3 [10]. We confirmed that knocking down HER3 in T47D cells with HER3^T355I^ and HER3^WT^ suppressed ERα expression. Knockdown of HER3 has also been reported to reduce ERα levels in MCF7 cells [6]. The exogenous expression of the HER3 T355I mutation results in increased activated HER3. How cells have elevated ERα levels upon activated HER3 requires further investigation.

Lapatinib is a dual tyrosine kinase inhibitor targeting both EGFR and HER2. Prickett *et al.* showed that lapatinib suppressed HER4 activation in melanoma [31]. Our immunoblotting data indicate that lapatinib treatment downregulated HER3^T355I^ activated HER4/HER1 expression in ER+ breast cancer cells. Lapatinib also blocked ERK1/2 activation, indicating that ERK1/2 is downstream of HER1/4-mediated signaling in ER+ cells. Cells expressing HER3^T355I^ when treated with fulvestrant or lapatinib suppressed cyclinD1 expression. Inhibition of ERK1/2 expression suppressed cyclinD1 expression which indicated that cyclinD1 mediated signaling downstream of the MAPK pathway. Also, blocking HER1/HER4 using lapatinib inhibited cyclinD1 expression. Overall, we propose a mechanism of activation of HER pathway in ER+ T47D and MCF-7 cells expressing HER3^T355I^ in MAPK(ERK1/2)-dependent cyclinD1-mediated pathway that triggered its proliferative potential (Figure 4E). Targeting HER3 using siRNA suppressed p-ERK1/2 and cyclinD1 expression indicating that HER3 could be a prominent therapeutic target for breast cancer cells with oncogenic HER3 mutations.

Based on structural modeling, the tethered threonine 355 is accommodated in a polar pocket within the domain II/III hinge in the inactive tethered conformation of HER3 ECD. Interactions between T355 and residues of the hinge region pocket (K329, V390, R391) are expected to stabilize HER3 in the inactive, tethered conformation by restraining the receptor from rotating around this hinge in the absence of ligand-dependent activation (Figure 4C). The bulkier, hydrophobic isoleucine residue at position 355 (I355) is expected to favor transition to the untethered, active conformation of HER3 ECD, thus promoting HER3 dimerization with other HER receptors and signaling even in the absence of a HER3 ligand (Figure 4D). Interestingly, T355 is conserved only in HER family members known to undergo ligand-mediated activation. As such, the equivalent residue in the orphan HER receptor, HER2, is subsituted by a lysine (K368).

HER3 mutants (F94L, G284R, D297Y, T355I and E1261A) demonstrated a gain-of function phenotype in MCF10AHER2 cells. Although AKT/MAPK pathways were not significantly activated in most HER3 mutants, we propose that the presence of significant EGFR in MCF10A might trigger signaling leading to their induced cell proliferation. Our immunoprecipitation data indicates that HER3 mutants (G284R, D297Y, T355I and E1261A) form heterodimers with HER2. Pertuzumab and Trastuzumab, monoclonal antibodies directed against HER2, were not effective in suppressing the proliferative potential of WT HER3. Another publication found that trastuzumab is mostly ineffective at suppressing matrigel acini formation using the MCF10AHER2 model [32].

Neratinib, a TKI directed against HER1/HER2/HER4 has recently been approved by the Food and Drug Administration (FDA) for adjuvant treatment of patients with early stage HER2-overexpressed/amplified breast cancer. Neratinib was given to 16 patients harboring HER3 gene mutations as a part of the SUMMIT trial. No clinical activity was observed in this HER3 mutant cohort which included HER3 mutations G284R, D297Y (*n =* 4) and T355A [33]. One explanation for the absence of clinical activity in these tumors is the possibility that these HER3 mutations could be passenger mutations, although our *in vitro* data for HER3 mutants G284R and D297Y does not support this. Another report similarly found that HER3 G284R and D297Y in the presence of HER2 to be oncogenic [18]. The amount of EGFR, HER2 or HER4 present in the patient tumors’ harboring HER3 mutations will further influence sensitivity to neratinib and influence the oncogenic capacity of HER3 mutations. Furthermore, the concentration of neratinib achieved in patient tumors will dictate the efficacy of neratinib. Our data indicated that 50 nM neratinib was not significantly effective to suppress the matrigel acini formation induced by most HER3 mutants indicating that a higher concentration of neratinib (500 nM) was necessary to abrogate the oncogenic potential of most HER3 mutants in MCF10AHER2 cells.

Suppressing HER3 expression in cancer cells which harbor endogenous HER3 mutations reduced MAPK (ERK1/2) signaling but not AKT, indicating that HER3 mutants may preferentially activate MAPK signaling over AKT-dependent mechanisms. Our study delineated a mechanism by which HER3 mutants drive their oncogenic signal in HER2+ MCF10A and ER+ T47D and MCF-7 breast cancer cell lines. We observed the HER3 mutation T355I to be oncogenic in the absence of HER2 over-expression in ER+ breast cancer, a novel finding and clinically relevant as this mutation has been identified in 4 ER+ breast cancer patients.

## MATERIALS AND METHODS

### Cell culture and inhibitors

Human breast cancer cells (T47D and MCF-7) were obtained from American Type Culture Collection (ATCC). SNU407, SNU1040 cells were purchased from Korean cell line bank. IGROV1 and HCT15 cell lines were obtained from NCI-Frederick Cancer DCTD tumor cell line repository. HEK293T cells were a generous gift from Dr. Susan Waltz, College of Medicine, University of Cincinnati. MCF10A cells overexpressing WT HER2 were generated in Dr. Carlos Arteaga’s lab, Department of Medicine, Vanderbilt-Ingram Cancer Center [34]. All cell lines were maintained in recommended media as per manufacturer’s guidelines. Neratinib and lapatinib were obtained from LC Laboratories. Fulvestrant was purchased from Sigma Aldrich. SCH772984 was obtained from Selleckchem. Pertuzumab and Trastuzumab were purchased from University of Cincinnati Medical Center Pharmacy.

### Sub-cloning and lentiviral production

HER3 mutations (F94L, G284R, D297Y, D313H, K329T, T355I, L792V, and E1261A) were introduced using site-directed mutagenesis. Mutant HER3 expression constructs were subcloned into the gateway-compatible lentiviral expression vector pDONR223-HER3 (Addgene). The expression constructs pDONER223-HER3 were subsequently subcloned into the gateway-compatible lentiviral pLX302 destination vector (Addgene). We confirmed the presence of HER3 gene by digestion with BsrGI restriction enzyme (data not shown). Lentiviral supernatants were obtained from HEK293T cells per manufacturer’s instructions. Infected MCF10AHER2, MCF-7 and T47D cells were selected using 2 and 0.5 µg/ml of puromycin (Santa Cruz Biotechnology) respectively.

### Small-interfering RNA

5x10^4^ cells (IGROV1, HCT15, SNU407, SNU1040 and T47D) were transfected with siRNAs specifically targeting HER3 (siHER3) or control siRNA (siCon). To knock down HER3 expression, siRNA against a HER3 target sequence ACCACGGTATCTGGTCATAAA was used [13] (Dharmacon International). Mismatched siRNA with a target sequence of GGAAGCAGACTCACTCTTATA was used as a negative control.

### Immunoblot analyses

ER+ cells (T47D and MCF-7) and MCF10AHER2 cells expressing HER3^EV^, HER3^WT^ and HER3 mutants (F94L, G284R, D297Y, D313H, K329T, T355I, L792V, and E1261A) were serum starved, lysed in RIPA buffer (ThermoFisher Scientific, Cat# BP-115) supplemented with protease and phosphatase inhibitors (ThermoFisher Scientific, Cat# 88669). In other experiments, ER+ cells with HER3^WT^ and HER3^T355I^ expression were serum starved and treated with vehicle (DMSO), lapatinib (1 µM) in presence or absence of fulvestrant (1 µM) or SCH772984 (1 µM) for 4 hr, cells were lysed and processed for immunoblotting. Furthermore, T47D cells with HER3^WT^ and HER3^T355I^ expression were transfected with siHER3 and siCon, cell lysates were collected after 48 hr. Ovarian (IGROV-1) and colorectal cancer cell lines (HCT-15, SNU-407, SNU-1040) were transfected and lysed under similar conditions. Serum starved MCF10AHER2 cells with HER3^EV^, HER3^WT^ and HER3 mutants were treated with 1 µM lapatinib for 0-4 hr, cells were lysed and processed for western blotting. Immunoblotting was performed using following antibodies: V5 (Cell Signaling Technology, Cat# 13202), ERα (Cell Signaling Technology, Cat# 8644), p-HER3 Tyr1289 (Cell Signaling Technology, Cat# 4791), p-HER3 Tyr1197 (Cell Signaling Technology, Cat# 4561), HER3 (Cell Signaling Technology, Cat# 12708), p-AKT S473 (Cell Signaling Technology, Cat# 4060), p-AKT Thr308 (Cell Signaling Technology, Cat# 9275), AKT (Cell Signaling Technology, Cat# 9272), p-ERK1/2 Thr202/Tyr204 (Cell Signaling Technology, Cat# 9101), ERK1/2 (Cell Signaling Technology, Cat# 9102), p-HER2 Tyr1221/1222 (Cell Sigaling Technology, Cat# 2243), HER2 (Cell Signaling Technology, Cat# 2165), p-EGFR/HER1 Tyr1068 (Cell Signaling Technology, Cat# 2234), EGFR/HER1 (Cell Signaling Technology, Cat# 4267), p-HER4 Tyr1284 (Cell Signaling Technology, Cat# 4757), HER4 (Cell Signaling Technology, Cat# 4795), CyclinD1 (Cell Signaling Technology, Cat# 2978), p-Tyr 1000 (Cell Signaling Technology, Cat# 8954) and Actin (Santa Cruz Biotechnology, Cat# SC-1616). Peroxidase-conjugated secondary antibodies (Santa Cruz Biotechnology) were used and protein signals were detected using Pierce ECL western blotting substrate (ThermoFisher Scientific, Cat# 32106).

### RTK array analysis

T47D and MCF-7 cells with HER3^WT^ and HER3^T355I^ expression (2 × 10^6^ cells/plate) were seeded in 100 mm plates (ThermoFisher Scientific), cultured for 24 hr and serum starved overnight. Cells were lysed and 300–600 µg proteins were used for human RTK and phospho kinase array (R&D Systems) as per manufacturer’s instructions.

### Cell viability assay

Growth kinetics of MCF10AHER2 cells with HER3 expression (EV, WT and mutants) was determined by MTT (4,5-dimethylthiazol-2-yl)-2,5-diphenyltetrazolium bromide) assay. Briefly, 5 × 10^3^ cells/well were seeded in 96 well plates (ThermoFisher Scientific) in triplicate. After specific time periods, media was substituted with 5 mg/ml MTT solution (Sigma Aldrich) and absorbance was recorded at 570 nm using SPECTRAmax PLUS Microplate Spectrophotometer Plate Reader (Molecular Devices Corporation) and expressed as the mean of triplicates relative to EV control together with standard error of mean. In separate experiments, ovarian (IGROV-1) and colorectal cancer cells (HCT-15, SNU407, SNU-1040 cells) were transfected using HER3 siRNA or control siRNA and reseeded (5 × 10^3^ cells/well). The cell proliferation was analyzed after 24 hr and represented as mean of triplicate values relative to respective consiRNA controls. The bar graph were generated using graph pad prism 7 (GraphPad Software, Inc., La Jolla, CA). In separate experiments, T47D and MCF-7 cells (5 × 10^3^ cells/well) expressing HER3^WT^ and HER3^T355I^ were seeded in triplicate and treated with vehicle (DMSO), lapatinib (0.25–1 µM) with or without fulvestrant (0.25–1 µM) and SCH772984 (0.25–1 µM) for 48 hrs and proceeded as above to obtain the CI values.

### Cell proliferation assay

MCF10AHER2, T47D and MCF-7 cells expressing HER3 (EV, WT and mutants) at a density of 5 × 10^4^– 5 × 10^5^ cells/well were seeded in 6 well plates (ThermoFisher Scientific) in triplicate. Complete media with 2 and 0.5 µg/ml of puromycin (Santa Cruz Biotechnology) respectively was replaced every alternate day and cells were stained with crystal violet in methanol within 5–25 days as described [35]. In separate experiments, ER+ cells with HER3^WT^ and HER3^T355I^ expression were treated with vehicle (DMSO), 1 µM lapatinib in the presence or absence of 1 µM fulvestrant or 1 µM SCH772984. Further, MCF10AHER2 cells expressing HER3 (EV, WT and mutants) were treated with vehicle (DMSO) ± 1 µM lapatinib. Media with each of the above inhibitors were changed every alternate day and stained within 5–15 days. In separate experiments, ovarian and colorectal cancer cell lines were transfected with siHER3 or siCon and reseeded in triplicate in 6 well plates and stained with crystal violet in methanol within 2–4 days after visible difference was noticed. The intensities were measured using Odyssey infrared System. The values were expressed as mean of intensities obtained from three independent experiments and bar graphs were generated using graph pad prism 7 (GraphPad Software, Inc., La Jolla, CA).

### Structural modeling

Structure-guided modeling to visualize the putative effect of the T355I mutation on the HER3 inactive state was performed in PyMOL (The PyMOL Molecular Graphics System, Version 2.0 Schrödinger, LLC) using the mutagenesis tool and the structure of the inactive HER3 extracellular domain (PDB ID: 1M6B) as a template [3]. Residue T355 was mutated to favored rotamers of isoluecine based on the penultimate rotamer library [36] and the top rotamer selected for structural analysis. All figures illustrating the T355I environment and HER3 activation mechanism were prepared in PyMOL (The PyMOL Molecular Graphics System, Version 2.0 Schrödinger, LLC) and Chimera [37].

### Immunoprecipitation

For immunoprecipitation (IP) experiments, stably transfected MCF10AHER2 cells expressing HER3 WT and mutant (G284R, D297Y, T355I and E1261A) were grown till 70% confluency and lysed in 1% NP40 lysis buffer containing 20 mM Tris, pH 7.4, 150 mM NaCl, 10% glycerol, 1 mM EDTA, 1 mM EGTA, 5 mM NaPyro-PO4, 50 mM NaF, 10 mM β-glycero-PO4. 700 micrograms (µg) of cleared lysate was incubated with 2 µg of V5 antibody (Cell Signaling Technology, Cat# 13202) overnight at 4° C. The next day, 5 µl Protein G magnetic beads (Millipore) were added per 1 µg of antibody and incubated for 1 hr at 4° C. Resuspended beads were washed with lysis buffer and boiled in SDS-PAGE loading buffer. The levels of V5, p-Tyr and HER2 from immunoprecipitated samples were analyzed by western blotting.

### Matrigel colony formation

Three dimensional (3D) growth assays were performed in growth factor-reduced matrigel (BD Biosciences) where 96 well plates were coated with 80 µl of matrigel/well. MCF10AHER2, T47D and MCF-7 (1 × 10^3^) cells expressing HER3 WT and mutants were plated and incubated at 37° C for 24 h. MCF10AHER2 cells with HER3^WT^ and mutants (F94L, G284R, D297Y, T355I, E1261A) were treated with with vehicle (DMSO), 1 µM lapatinib or 50 and 500 nM neratinib every alternate day. In separate experiments, MCF10AHER2 cells with HER3^WT^ and mutants were treated with pertuzumab (30 ug/mL) ± trastuzumab (20 ug/mL) under similar conditions. T47D and MCF-7 cells with HER3^WT^ and HER3^T355I^ expression were treated with vehicle (DMSO), lapatinib (1 µM), in presence or absence of 1 µM fulvestrant or 1 µM SCH772984. Existing media was replenished with vehicle and drugs every other day. After 10 days of incubation, colonies were visualized and photographs were captured from 3 random fields from each well under microscope (Nikon) at 10× magnification. The areas were measured by ImageJ and expressed as % of control treatments. The bar graphs were generated using graph pad prism 7 (GraphPad Software, Inc., La Jolla, CA). The experiments were repeated three times to confirm the results.

### Statistical analysis

Data are shown as the mean ± standard error of mean (SEM) and representative of at least three independent experiments. Statistical analysis among groups using the two-tailed Student’s *t*-test, one-way analysis of variance, *P <* 0.05 was considered statistically significant. The combination index (CI) was obtained using compuSyn software.

## SUPPLEMENTARY MATERIALS FIGURES AND TABLE


