# Supplementary material for: Activating HER3 mutations in breast cancer
Source: Oncotarget. 2018 Jun 12;9(45):27773–88. doi: 10.18632/oncotarget.25576 (PMC6021238; doi:10.18632/oncotarget.25576)
Supplement: Supplementary file 1 [file oncotarget-09-27773-s001.pdf]

# Activating HER3 mutations in breast cancer

## SUPPLEMENTARY MATERIALS

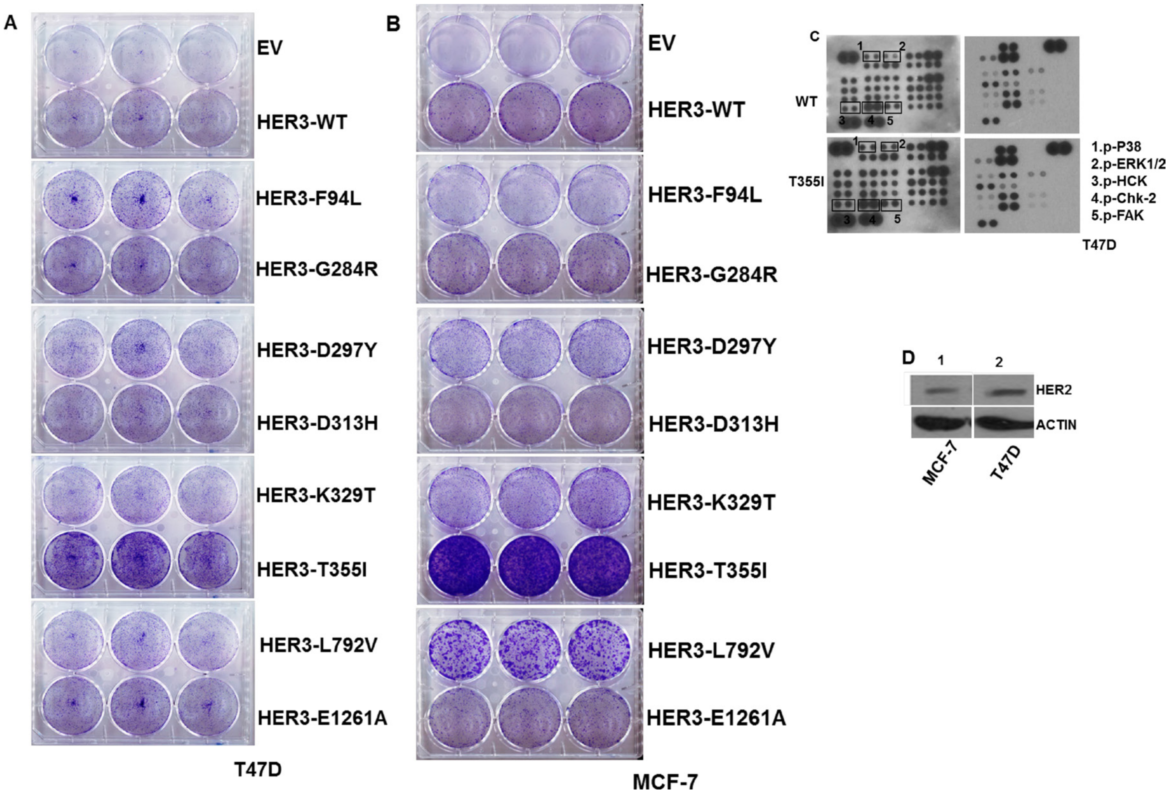


**Supplementary Figure 1: Proliferative potential of ER+ cells expressing HER3 mutants.** (**A–B**) Representative stained plates of ER+ cells (T47D and MCF-7) expressing HER3$^{EV}$, HER3$^{WT}$ and HER3 mutants (**C**) Phosphokinase array was performed to assess kinase phosphorylation of various intracellular kinases in T47D (HER3$^{WT}$ and HER3$^{T355I}$) cell lysate. (**D**) HER2 expression is analyzed in ER+ T47D and MCF-7 cells using western blotting. Actin served as loading control.

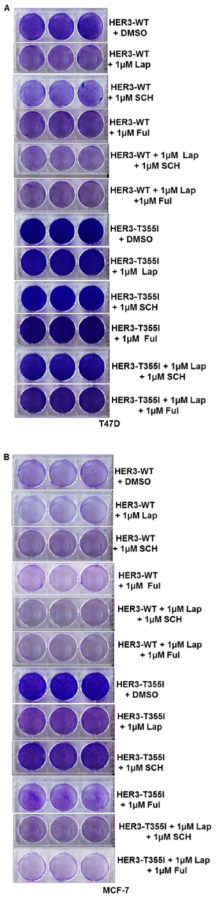


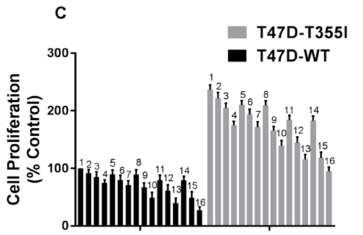


| T47DWT | | | | | | | | | | | | | | | | |
|---|---|---|---|---|---|---|---|---|---|---|---|---|---|---|---|---|
| **COLUMN NO.** | **1** | **2** | **3** | **4** | **5** | **6** | **7** | **8** | **9** | **10** | **11** | **12** | **13** | **14** | **15** | **16** |
| **LAP (µM)** | - | 0.25 | 0.5 | 1 | - | - | - | - | - | - | 0.25 | 0.5 | 1 | 0.25 | 0.5 | 1 |
| **SCH (µM)** | - | - | - | - | 0.25 | 0.5 | 1 | - | - | - | 0.25 | 0.5 | 1 | - | - | - |
| **FUL (µM)** | - | - | - | - | - | - | - | 0.25 | 0.5 | 1 | - | - | - | 0.25 | 0.5 | 1 |
| | | | | | | | | | | **CI** | 0.9 | 0.5 | **0.71** | 0.78 | 0.58 | **0.61** |

| T47DT355I | | | | | | | | | | | | | | | | |
|---|---|---|---|---|---|---|---|---|---|---|---|---|---|---|---|---|
| **COLUMN NO.** | **1** | **2** | **3** | **4** | **5** | **6** | **7** | **8** | **9** | **10** | **11** | **12** | **13** | **14** | **15** | **16** |
| **LAP (µM)** | - | 0.25 | 0.5 | 1 | - | - | - | - | - | - | 0.25 | 0.5 | 1 | 0.25 | 0.5 | 1 |
| **SCH (µM)** | - | - | - | - | 0.25 | 0.5 | 1 | - | - | - | 0.25 | 0.5 | 1 | - | - | - |
| **FUL (µM)** | - | - | - | - | - | - | - | 0.25 | 0.5 | 1 | - | - | - | 0.25 | 0.5 | 1 |
| | | | | | | | | | | **CI** | 0.46 | 0.3 | **0.31** | 0.67 | 0.32 | **0.42** |

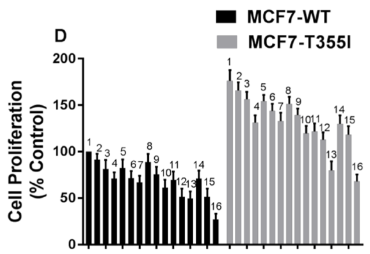


| MCF-7WT | | | | | | | | | | | | | | | | |
|---|---|---|---|---|---|---|---|---|---|---|---|---|---|---|---|---|
| **COLUMN NO.** | **1** | **2** | **3** | **4** | **5** | **6** | **7** | **8** | **9** | **10** | **11** | **12** | **13** | **14** | **15** | **16** |
| **LAP (µM)** | - | 0.25 | 0.5 | 1 | - | - | - | - | - | - | 0.25 | 0.5 | 1 | 0.25 | 0.5 | 1 |
| **SCH (µM)** | - | - | - | - | 0.25 | 0.5 | 1 | - | - | - | 0.25 | 0.5 | 1 | - | - | - |
| **FUL (µM)** | - | - | - | - | - | - | - | 0.25 | 0.5 | 1 | - | - | - | 0.25 | 0.5 | 1 |
| | | | | | | | | | | **CI** | 0.58 | 0.4 | **0.35** | 0.62 | 0.58 | **0.46** |

| MCF-7T355I | | | | | | | | | | | | | | | | |
|---|---|---|---|---|---|---|---|---|---|---|---|---|---|---|---|---|
| **COLUMN NO.** | **1** | **2** | **3** | **4** | **5** | **6** | **7** | **8** | **9** | **10** | **11** | **12** | **13** | **14** | **15** | **16** |
| **LAP (µM)** | - | 0.25 | 0.5 | 1 | - | - | - | - | - | - | 0.25 | 0.5 | 1 | 0.25 | 0.5 | 1 |
| **SCH (µM)** | - | - | - | - | 0.25 | 0.5 | 1 | - | - | - | 0.25 | 0.5 | 1 | - | - | - |
| **FUL (µM)** | - | - | - | - | - | - | - | 0.25 | 0.5 | 1 | - | - | - | 0.25 | 0.5 | 1 |
| | | | | | | | | | | **CI** | 0.37 | 0.6 | **0.5** | 0.59 | 0.8 | **0.48** |

**Supplementary Figure 2: Blocking of HER4/HER1 and ERK1/2/ER signaling inhibits the proliferation of ER+ cells with HER3$^{T355I}$.** (**A**) T47D cells expressing HER3$^{WT}$ and HER3$^{T355I}$ were plated in 6 well plates and treated with vehicle (DMSO), lapatinib (1 µM), fulvestrant (1 µM), SCH772984 (1 µM) ± indicated combinations. Representative image of stained plate of T47D (HER3$^{WT}$ and HER3$^{T355I}$) cells ± indicated combinations. (**B**) Representative image of stained plate of MCF-7 cells with HER3$^{WT}$ and HER3$^{T355I}$ overexpression treated with vehicle (DMSO), fulvestrant (1 µM), lapatinib (1 µM), SCH772984 (1 µM) ± indicated combinations. (**C**–**D**) ER+ cells expressing HER3$^{WT}$ and HER3$^{T355I}$ were plated in triplicate and treated with vehicle (DMSO), lapatinib (0.25–1 µM) ± SCH772984 (0.25–1 µM) and lapatinib (0.25–1 µM) ± fulvestrant (0.25–1 µM) for 48 hrs and the growth was evaluated using the MTT assay. The value is represented as mean ± SEM ($n$ = 3 independent experiments performed in triplicate). Synergistic combinations are indicated by combination index (CI) values <1. The CI values for 1 µM lapatinib ± 1 µM SCH772984 and 1 µM lapatinib ± 1 µM fulvestrant are highlighted in bold.

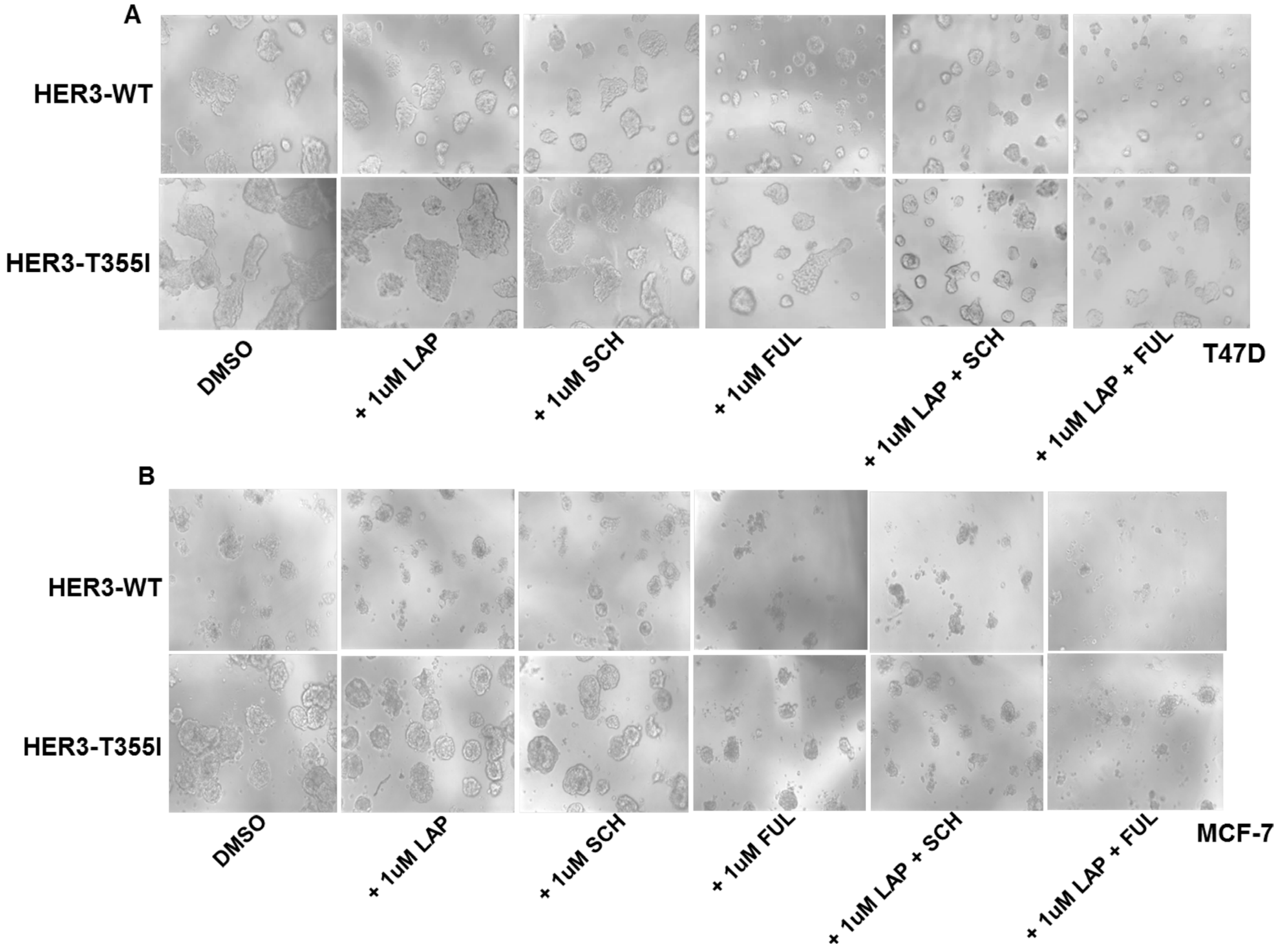


**Supplementary Figure 3:** (**A**) Phase contrast images of colonies formed by T47D cells expressing $HER3^{WT}$ and $HER3^{T355I}$ on matrigel treated with vehicle (DMSO), fulvestrant (1 µM), lapatinib (1 µM), SCH772984 (1 µM) ± indicated combinations at 10× magnification. (**B**) Phase contrast images of matrigel colonies formed by MCF-7 cells expressing $HER3^{WT}$ and $HER3^{T355I}$ treated with vehicle (DMSO), fulvestrant (1 µM), lapatinib (1 µM), SCH772984 (1 µM) ± indicated combinations at 10× magnification.

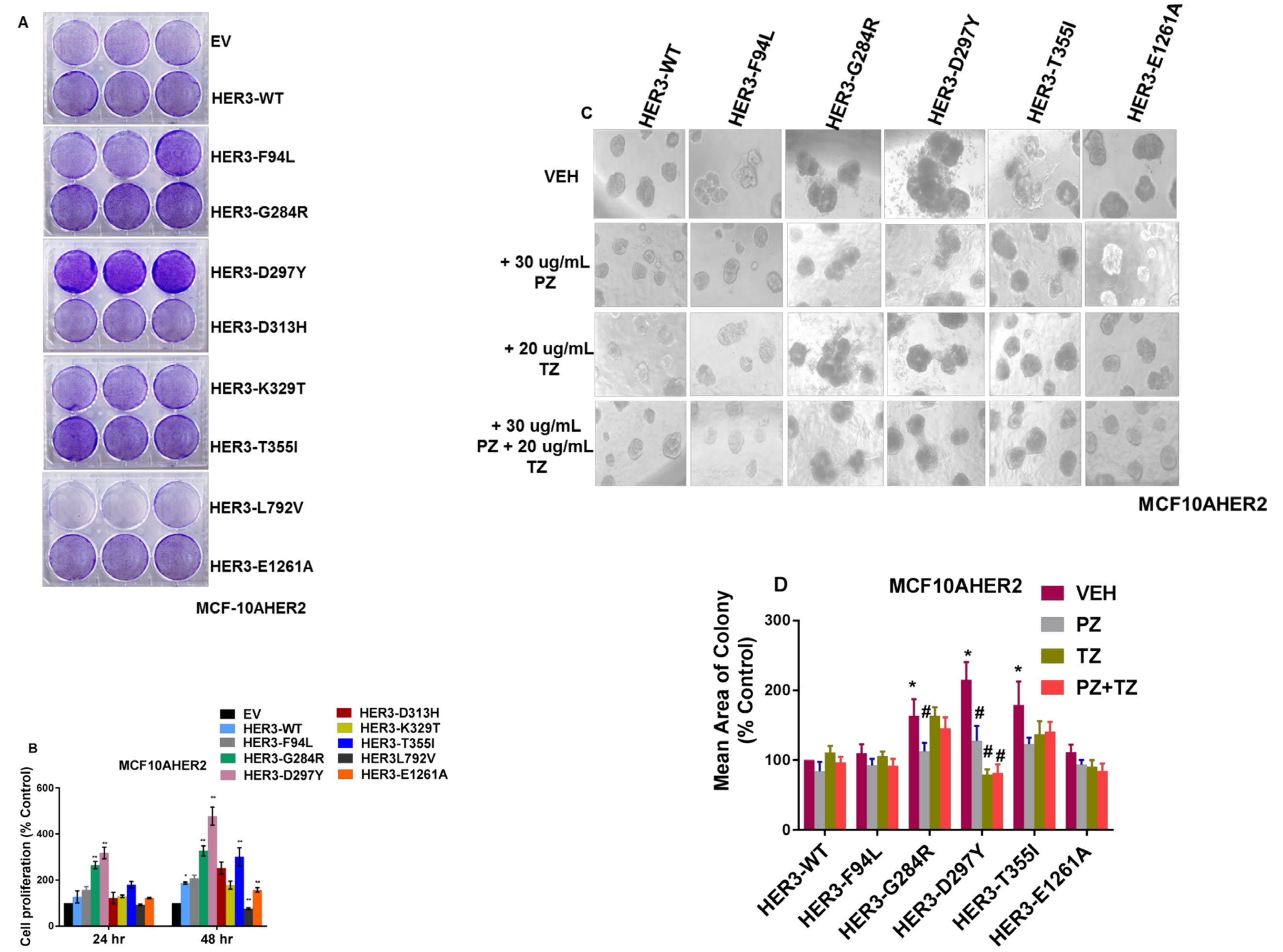


**Supplementary Figure 4: Oncogenic potential of MCF10AHER2 cells expressing HER3 mutants.** (**A**) Representative stained plates of MCF10AHER2 cells expressing HER3 mutants. (**B**) Growth kinetics of MCF10AHER2 cells with HER3 mutations was determined in vitro using the MTT assay for 24–48 hr and represented in form of bar graph. Error bars: SEM, ($n$ = 3, from three different independent experiments).$^{*}p < 0.05$ versus EV, $^{**}p < 0.05$ versus WT (**C**–**D**) MCF10AHER2 cells expressing HER3 WT and mutants were cultured in 3D matrigel in presence of vehicle (PBS), pertuzumab (PZ) and/or trastuzumab (TZ) at the indicated concentrations and treated every alternate day and photographed on day 10. The average size of each cellular structure was quantified using Image J software represented as percentage of control. Error bars: SEM (n=3, from three independent experiments in triplicate). $^{*}p < 0.05$ versus WT, $^{\#}p < 0.05$ versus respective control.

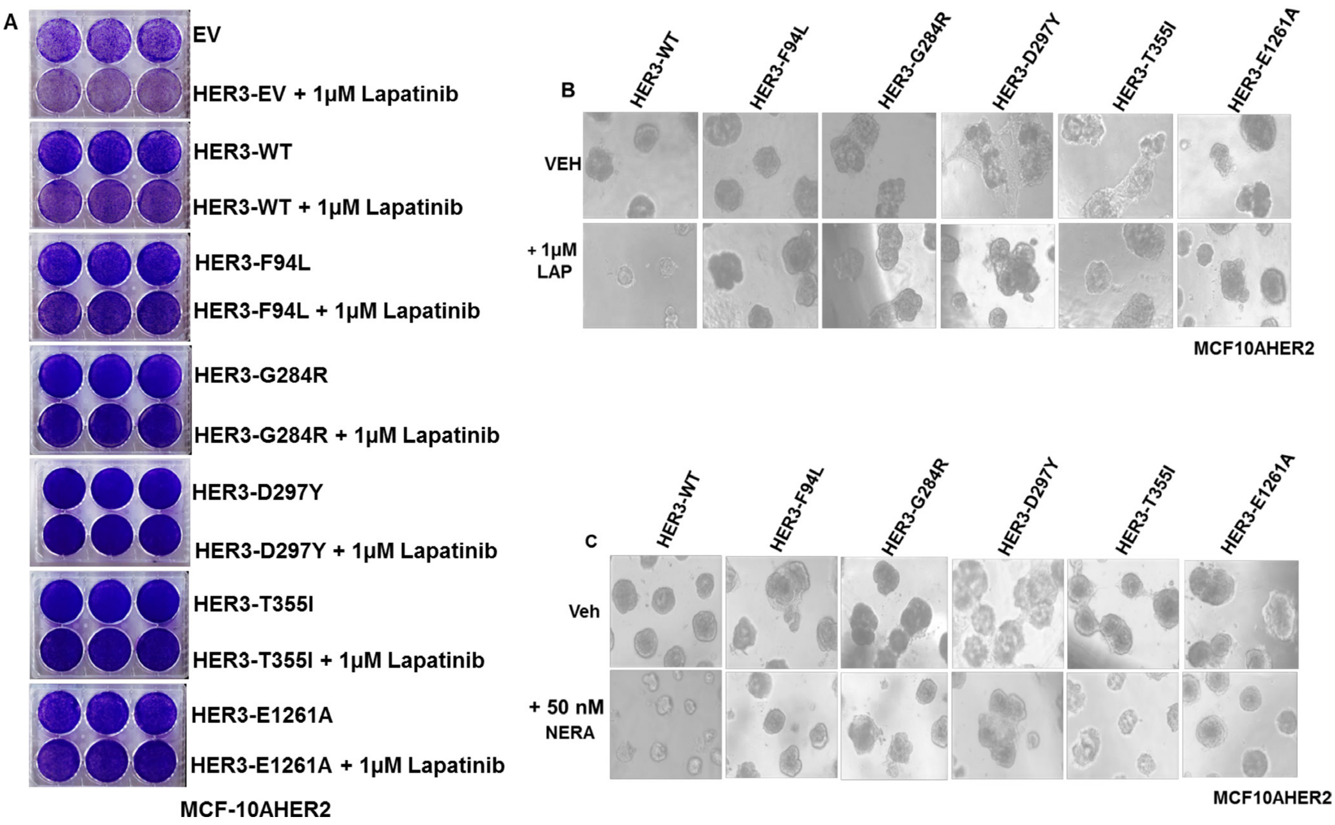


**Supplementary Figure 5: Effect of lapatinib and neratinib on MCF10AHER2 cells expressing HER3 mutants.** (**A**) Representative stained plate of MCF10AHER2 cells overexpressing HER3 EV,WT and mutants ± lapatinb. (**B**) Phase contrast images of matrigel colonies formed by MCF10AHER2 cells expressing $HER3^{WT}$ and HER3 mutants treated with vehicle (DMSO) ± 1 µM lapatinib at 10× magnification. (**C**) Phase contrast images of matrigel colonies formed by MCF10AHER2 cells expressing $HER3^{WT}$ and HER3 mutants treated with vehicle (DMSO) ± 50 nM neratinib at 10× magnification.

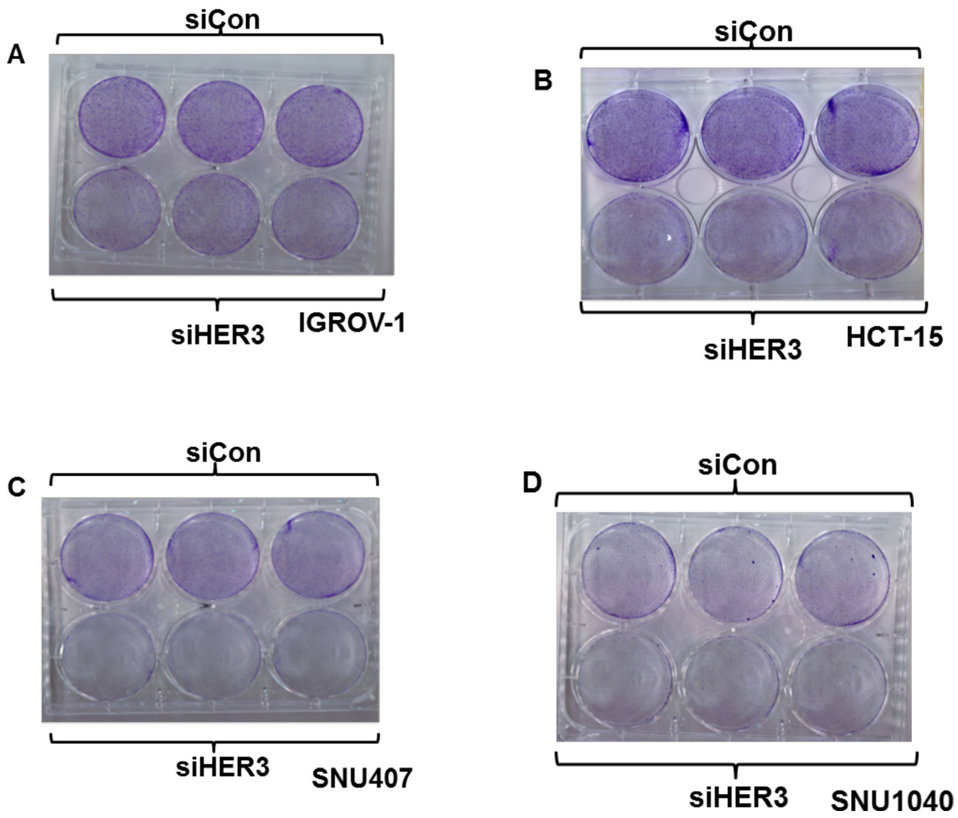


**Supplementary Figure 6: Knocking down HER3 suppresses cell proliferation of ovarian and colorectal cancers harboring endogenous HER3 mutations.** (**A**–**D**) Ovarian (IGROV1) and colorectal cancer cell lines (HCT15, SNU407, SNU1040) transfected with siHER3 or siCon, reseeded and stained. Representative images of the above stained plates.

**Supplementary Table 1: List of breast cancer patients' case IDs having HER3 mutations with hormone receptor status**

| Sr no | Case ID | Amino acid change | Cancer type | HER2/ER status | Technique to evaluate HER2/ER status |
|---|---|---|---|---|---|
| **1** | TCGA-C8-A12T | F94L | Breast invasive ductal carcinoma | HER2+/ER+ | HER2/ER: IHC |
| **2** | METABRIC-MB-0294 | G284R | Breast invasive ductal carcinoma | HER2+/ER+ | HER2: SNP6<br>ER: IHC |
| **3** | METABRIC-MB-2964 | G284R | Breast invasive ductal carcinoma | HER2+/ER+ | HER2: SNP6<br>ER: IHC |
| **4** | METABRIC-MB-4908 | G284R | Breast invasive ductal carcinoma | HER2+/ER+ | HER2:SNP6<br>ER: IHC |
| **5** | TCGA-B6-A0IK | D297Y | Breast invasive ductal carcinoma | HER2 enriched/ER- | HER2/ER: IHC |
| **6** | SC-HER2-068 | D313H | Metastatic breast carcinoma | HER2 enriched/ER+ | ER: IHC<br>HER2: CNA |
| **7** | SC-HER2-049 | K329T | Metastatic breast carcinoma | HER2 enriched/ER+ | ER: IHC<br>HER2: CNA |
| **8** | METABRIC-MB-5284 | T355I | Breast invasive ductal carcinoma | HER2-/ER+ | HER2:SNP6<br>ER: IHC |
| **9** | METABRIC-MB-4992 | T355I | Breast invasive lobular carcinoma | HER2-/ER+ | HER2:SNP6<br>ER: IHC |
| **10** | TCGA-BH-A18P | T355I | Breast mixed ductal and lobular carcinoma | HER2+/ER+ | HER2: IHC& FISH<br>ER: IHC |
| **11** | TCGA-LL-A5YL-01 | T355I | Breast mixed ductal and lobular carcinoma | HER2+/ER+ | HER2: IHC&FISH<br>ER: IHC |
| **12** | MTS-T2405 | T355I | Invasive breast carcinoma | Unknown | N/A |
| **13** | TCGA-A8-A09G | L792V | Breast mixed ductal and lobular carcinoma | HER2+/ER+ | HER2: IHC& FISH<br>ER: IHC |
| **14** | TCGA-AN-A0FV | E1261A | Breast invasive ductal carcinoma | HER2+/ER− | HER2/ER: IHC |

Abbreviations: IHC: Immunohistochemistry, SNP: Single nucleotide polymorphism, FISH: Fluorescence in situ hybridization, CNA: Copy number analysis [1].

## REFERENCES

1. Toy W, Shen Y, Won H, Green B, Sakr RA, Will M, Li Z, Gala K, Fanning S, King TA, Hudis C, Chen D, Taran T, et al. ESR1 ligand-binding domain mutations in hormone-resistant breast cancer. Nat Genet. 2013. https://doi.org/10.1038/ng.2822.
